# Plagiarism: word, idea, figure, etc

**DOI:** 10.3325/cmj.2011.52.657

**Published:** 2011-10

**Authors:** Viroj Wiwanitkit

**To the Editor:** The recent publication by Habibzadeh and Shashok discussed the importance of direct verbatim textual plagiarism (1). Indeed, plagiarism can be found in many forms, including textual, conceptual, and figure plagiarism (2). However, it is important to realize and understand what is considered plagiarism and what is not. In medicine, the use of a concept proposed by others is very common and this might not be considered plagiarism if the author acknowledges the use by proper referencing and citation. Paraphrasing with adequate referencing and citation can also be acceptable. Many plagiarism detection tools might not be useful in detection of all forms of plagiarism but they still are a tool for detection of simple verbatim plagiarism and are better than having no tool to fight the plagiarist.
